# The Threat of Disruptive Jamming to Blockchain-Based Decentralized Federated Learning in Wireless Networks

**DOI:** 10.3390/s24020535

**Published:** 2024-01-15

**Authors:** Gyungmin Kim, Yonggang Kim

**Affiliations:** 1 Agency for Defense Development, Daejeon 34186, Republic of Korea; gmkim@add.re.kr; 2 Division of Computer Science and Engineering, Kongju National University, Cheonan 31080, Republic of Korea

**Keywords:** blockchain, federated learning, jamming attack

## Abstract

Machine learning techniques have attracted considerable attention for wireless networks because of their impressive performance in complicated scenarios and usefulness in various applications. However, training with and sharing raw data obtained locally from each wireless node does not guarantee privacy and requires a large communication overhead. To mitigate such issues, federated learning (FL), in which sharing parameters for model updates are shared instead of raw data, has been developed. FL has also been studied using blockchain techniques to efficiently perform learning in distributed wireless systems without having to deploy a centralized server. Although blockchain-based decentralized federated learning (BDFL) is a promising technique for various wireless sensor networks, malicious attacks can still occur, which result in performance degradation or malfunction. In this study, we analyze the impact of a jamming threats from malicious miners to BDFL in wireless networks. In a wireless BDFL system, it is possible for malicious miners with jamming capability to interfere with the collection of model parameters by normal miners, thus preventing the victim miner from generating a global model. By disrupting normal miners participating in BDFL systems, malicious miners with jamming capability can more easily add malicious data to the mainstream. Through various simulations, we evaluated the success probability performance of malicious block insertion and the participation rate of normal miners in a wireless BDFL system.

## 1. Introduction

Along with the rapid development and impressive performance of machine learning (ML) techniques, many wireless network researchers have adopted ML-based solutions to overcome optimization problems in unknown or highly complicated environments [1]. However, conventional data-driven ML algorithms require large communication overheads to transmit raw data from multiple mobile nodes to a centralized ML processor. In addition, even if mobile edge nodes are equipped with the computational capacity to train a local learning model, data sharing with neighboring nodes is still required because the amount of data that each device can collect is limited. More importantly, in this type of learning process, there are concerns about security and privacy during data transmission and sharing [2].

Recently, collaborative learning, called federated learning (FL), has attracted much attention. FL-based algorithms perform the training process without exchanging local data samples [3,4]. A centralized server coordinates edge nodes participating in FL and produces a global model using parameters collected from locally trained models. After producing the global model, edge nodes download the globally updated model and compute the next local update. However, because of closed-loop model exchanges, the delay of each global model update is occasionally more than several minutes (e.g., 10 min or more in some systems) [3]. In addition, connectivity can be unstable and difficult to guarantee when the communication channel is vulnerable or malfunction occurs at the centralized server [5].

Pokhrel and Choi [6] proposed decentralized FL that exploits the blockchain-based model update and verification process in a distributed manner to coordinate the global model. Blockchain-based decentralized FL (BDFL) can provide privacy for the nodes by sharing the model parameters instead of raw data, and they work in a decentralized manner using the blockchain mechanism. By providing rewards proportional to the usefulness of data sample sizes, BDFL motivates nodes to actively participate in learning [6]. In addition, BDFL can extend the usage scenarios of FL in wireless networks such as unmanned aerial vehicle networks [7]. When BDFL is adopted in a wireless network system, the collection/sharing of locally updated models and the broadcasting of new blocks are transmitted through wireless channels. However, due to the vulnerability of wireless networks channels, security issues caused by jamming attacks still exist. Wireless channel data transmission can be severely affected by interference signals and fail when the signal-to-interference-plus-noise ratio (SINR) is insufficient. Therefore, when there are strong jamming signals, miners may fail to receive any local parameters and be excluded from the BDFL system.

If the number of participating miners decreases because of a jamming attack, the malicious miner group can achieve a higher attack success probability even with the same computing power. In this study, we investigated the impact of jamming attacks on BDFL in wireless networks. We assumed that the malicious miner group has a jamming capability and attempts to insert a malicious block into the ledgers of normal miners. In a wireless BDFL system, miners associated with adjacent edge nodes collect the parameters of locally updated models and perform block mining to generate a new block with its updated global model. A new miner block that successfully generates the hash is propagated to other miners. However, data transmission from edge nodes is vulnerable to interference signals. By exploiting this vulnerability in a BDFL system, malicious miners participating in the mining process of the blockchain system interfere with other miners’ data collection by jamming signals. Because normal miners that fail to receive sufficient local model parameters do not perform proof-of-work computation, malicious miners can exclude other miners from BDFL by the jamming attack. Then, malicious miners can succeed in proof-of-work computation with a higher probability even with the same computing power, thus inserting malicious blocks into the mainstream. In other words, the malicious miner group can perform a 51% attack without a significant increase in the computing power required for block mining. When a group of miners controls more than 50% of the network’s mining hash rate, they can control the blockchain mainstream. In this way, the attacker group can introduce an altered blockchain into the network, called a 51% attack or majority attack [8]. In this paper, we describe the outage probability caused by jamming attacks and the corresponding attack success probability of attackers who attempt to add malicious blocks to the mainstream. The main contributions of this study are summarized as follows:

- We present a security threat that can be induced by a malicious miner group with jamming capabilities in blockchain-based FL operating in a wireless network. Under proof-of-work mechanisms for blockchain consensus protocols, normal miners calculate a hash value to upload a global model generated by combining local model parameters received from adjacent edge nodes to the blockchain. If sufficient data cannot be collected because of a jamming attack, the corresponding miner will not proceed with the mining process and be excluded from BDFL. In addition, we present a conceptual countermeasure to respond to the attack.
- We analyze the outage probability of normal miners and the success probability of malicious block insertion under jamming attacks. Jamming attacks increase the outage probability of normal miners, and as normal miners’ mining participation decreases, the block insertion success probability of malicious miner groups increases.
- Using various simulations, we evaluated the performance of the success probability of malicious block insertion and the participation rate of normal miners in a wireless BDFL system.

This paper is organized as follows. In Section 2, we present related work on blockchain-based FL and its security issues including jamming attacks in wireless networks. In Section 3, a system model for the considered BDFL system in a wireless network and signal transmissions under a jamming attack by malicious miners is described. In Section 4, we analyze the outage probability of normal miners and the success probability of attacks that attempt to insert malicious blocks into the mainstream of BDFL systems. In Section 5, the simulation results are presented, and the conclusion is presented in Section 6.

## 2. Related Work

ML is a data-based learning algorithm, and the amount of data used for training directly affects learning performance. In conventional learning algorithms, a system in which a large amount of data are aggregated and used on a centralized server has been considered [9]. However, data aggregation on a centralized server is problematic for guaranteeing privacy [2]. FL was proposed to prevent privacy issues and alleviate system overheads during data transmission and sharing. FL aggregates local model parameters instead of data samples originating from each edge node [4,10]. In FL, a centralized server produces a global model using the parameters collected from locally trained models, and edge nodes download the globally updated model and compute their next local update.

Concerned about server malfunction and connection to the server, Pokhrel and Choi [6] addressed decentralized FL that manages a global model through a blockchain mechanism rather than deploying a centralized server or unit. They developed a mathematical framework that features a controllable network and blockchain-based FL parameters, such as the number of retransmissions, block sizes, block arrival rates, and frame sizes. End-to-end delay is quantified using the analysis results, and a delay minimization algorithm that adjusts the block arrival rate based on channel dynamics was proposed. Lu et al. [11] developed a secure data-sharing structure using blockchain technology, tailored for distributed stakeholders. They incorporated privacy-preserving FL into the consensus mechanism of a permission-based blockchain, enabling the computational efforts required for consensus to simultaneously facilitate federated training. Nguyen et al. [12] presented an overview of the fundamental concepts and explored the opportunities of integrating of FL and blockchain in multi-access edge computing networks. Cui et al. [13] studied a fast blockchain-based FL to handle large amounts of communication traffic in practical scenarios. They proposed a compression communication method for blockchain-based FL to improve communication efficiency. The proposed method compresses traffic while minimizing the training loss subject to a limited training time. They verified the performance of the proposed method with regard to compression and block generation rate. Ali et al. [14] conducted a comprehensive analysis of IIoT literature focusing on the integration of Blockchain and federated learning to augment intrusion detection systems and boost their capabilities in detecting threats. Beltrán et al. [15] examined the key differences between centralized and decentralized FL, focusing on federation architectures, topologies, communication methods, and security approaches.

Aponte-Novoa et al. [16] investigated 51% attacks using block mining behavior. By detailing the characterization of miners in the Bitcoin and Crypto Ethereum blockchain, they identified a mining pattern among the main miners. They also demonstrated that the hash rate power was increased in a minimal set of miners, which could present a risk to the current blockchain-based system. Ye et al. [8] proposed a tree-structure method that simulates the blockchain process and analyzes the relationship between the number of attacks and the number of states to evaluate the security of each state. They applied the 51% attack strategy to simulate the attacker’s behavior to determine the trend of the number of states and the number of attacks. Shrestha and Nam [17] investigated the design of a regional blockchain of VANETs to achieve a low 51% attack success probability. They derived a condition that guarantees a low 51% attack success probability in terms of the number of normal and malicious nodes, message delivery time, and puzzle computation time. Yang et al. [18] combined the history-weighted information of miners using the total calculation difficulty to increase the cost of a traditional attack to alleviate the 51% attack problem. Khan et al. [19] examined the X.509 Public-Key Infrastructure (PKIX) system, focusing on its architecture, history, certificate issuance, and vulnerability to cyber-attacks. They covered various PKIX and certificate revocation proposals, including their modern implementation on blockchain and ledger technologies. Guru et al. [20] investigated the current landscape of blockchain consensus algorithms, focusing on their security features and vulnerabilities to various attacks such as ARP, DDoS, and sharding in permissionless blockchains.

Along with the 51% attack problem, security issues regarding blockchains and FL have been studied. Gangwani et al. [21] proposed an architecture that involves various types of nodes that can be used to integrate IoT devices and blockchain technology. They identified and described various challenges that arise when a blockchain and IoT are integrated. Ruby et al. [22] investigated the challenges of selecting clients and allocating channels, as well as managing power control in the uplink process of FL within the IoMT field. This was particularly focused on scenarios involving a jammer, with an emphasis on the impact on the duration of long-term learning. They utilized the Stackelberg game to find the joint best response strategy for the jammer and FL network by leveraging the difference of convex programming approach and the dual decomposition technique. Xu et al. [23] investigated the security performance of wireless blockchain networks in the presence of malicious jamming when the Raft consensus mechanism is adopted. Shayan et al. [24] introduced a completely decentralized, peer-to-peer method for multi-party ML. This approach leverages blockchain technology and cryptographic elements to facilitate a privacy-secured ML procedure among peering clients. Shi and Sagduyu [25] studied how to launch over-the-air jamming attacks to disrupt the FL process when it is executed over a wireless network. They took into account the impact of jamming attacks on various transmission aspects: this included the disruption of local model updates sent from clients to the server, interference with global model updates transmitted from the server to clients, or a combination of both scenarios.

Research on jamming attacks continues to target vulnerabilities in the physical layer of wireless networks. Kim et al. [26] studied a deep learning-based reactive jamming attack that selectively transmits jamming signals when the detected signal is expected to be a control frame such as an acknowledge frame. Amuru et al. [27] studied a learning-based jamming attack that adaptively adjusts physical layer parameters. Their proposed method is based on a multi-armed bandit, and the signaling scheme, transmission power, and jamming duration are adjusted to maximize the jamming efficiency against stationary pairs of transmitter and receiver nodes. The attacker learns the duration of signals by training the deep neural networks using the front part of the collected signals and reactively attacks the transmitted signal in the air when the duration of the detected signal is expected to be shorter than the threshold. Kim and Lim [28] studied beamforming-based jamming attacks in blind networks. Assuming that attackers have no prior knowledge in blind networks, they proposed a reinforcement learning-based beamforming-based jamming attack that measures statistical changes in the channel busy time to evaluate the impact of the jamming. Pirayesh et al. [29] presented an extensive survey of existing jamming attacks and anti-jamming strategies across various wireless networks.

Although many recent studies have focused on security issues regarding blockchain and FL, consideration of the environment in which jamming attacks occur has not received much attention. H. M. Buttar et al. [30] investigated a comprehensive study aimed at mitigating active attacks on IoT networks, which leverage blockchain technology for enhanced security. They considered network model comprises a singular leader node responsible for the centralized management of logs and transactions, alongside various follower nodes. However, a notable gap persists in the existing literature regarding strategies to counter jamming attacks within BDFL. Because threats to the physical layer of wireless networks are inevitable, these vulnerabilities must be considered. To the best of our knowledge, the current paper is the first to address the threat of jamming attacks specifically within wireless systems employing BDFL. In this study, we analyzed a scenario in which a malicious miner group with jamming capability aims to increase the probability of block insertion success by interfering with the data collection of neighboring miners in the BDFL system. In our previous work, we presented a conceptual scenario in which malicious miners with jamming capabilities intervened in a BDFL system [31]. We provided a proof-of-concept of the impact of jamming on blockchain-based processes by simply limiting the computational power of normal miners. To improve this, herein, the attack on the BDFL system scenario and the system model are further specified, and the jamming impact and resulting block insertion success probability are analyzed.

## 3. System Model

**Figure 1:**
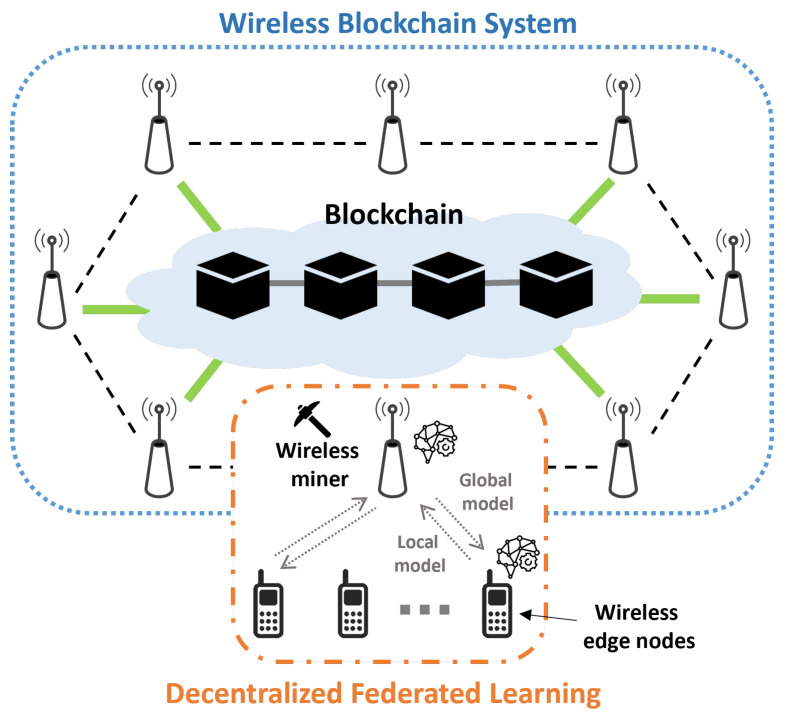
Blockchain-based decentralized federated learning in wireless networks.

We considered a wireless network scenario in which edge nodes can train their local learning model and upload/download learning parameters to/from an associated miner in a BDFL system. Figure 1 shows wireless network BDFL system. Each wireless edge node downloads a global model from the associated wireless miner that updates the global model by aggregating local model parameters from associated edge nodes or using the global model received in the new block from other miners that successfully generates the hash for the new block. Next, each edge node updates the local model with the help of the global model and trains the local model with newly obtained local data samples. Then, each miner collects locally updated model parameters from associated nodes and performs mining to generate a new block. When miners receive newly updated parameters, they broadcast the obtained parameters to other miners to accelerate the learning convergence and alleviate the biased learning. Note that the number of collected sets of model parameters for each miner can vary depending on the location of the miners, channel conditions, and number of associated nodes.

### 3.1. Blockchain-Based Decentralized FL in Wireless Networks

In the BDFL system, we denote M as the set of normal miners, where $M=\{m_{1},m_{2},\cdots ,m_{M}\}$ and *M* is the number of miners. Miner $m_{j}$ is connected to $N_{j}$ edge nodes, where $N_{j}=\left\{n_{j,1},n_{j,2},\cdots ,n_{j,N_{j}}\right\}$ is the set of edge nodes connected to $m_{j}$, and N is the set of all edge nodes. Miner $m_{j}$ performs block mining and collects local model parameters from $N_{j}$ and adjacent miners. $M_{j}=\left\{m_{j,1},m_{j,2},\cdots ,m_{j,M_{j}}\right\}$ denotes the set of miners within the communication range of $m_{j}$, where $M_{j}$ is the size of set $M_{j}$. Using the collected local model data, each miner generates a global model candidate which becomes the new global model when the corresponding miner successfully generates the hash for the new block. The collected local model parameters are aggregated in accordance with the adopted model aggregation algorithm (i.e., FedSGD and FedAvg).

Each miner inserts the collected local parameters into a contending block until the block size reaches the threshold $R_{s}$ or the gathering time exceeds the time threshold $R_{t}$. The miner then conducts the model aggregation. The miner then begins the block generation process, and the newly generated block is propagated into the blockchain system and verified if one miner successfully generates the hash for the new block. In this study, the blockchain system adopts a proof-of-work consensus mechanism that requires a significant amount of computing power from a network of devices. When a miner receives a newly generated block during its block generation, it stop the block generation computation and verifies the received block by checking the hash value. If the received new block has the correct hash value, the miner sends an acknowledgment and performs the mining process for the next block. All nodes are associated with their miner download a new global model and use it for local model updates.

In this study, we assumed that each miner sets out on a block generation process when the number of collected local model data in the contending block is *N*th or more. For the next global model, $m_{j}$ collects local model data from $N_{j}$ nodes and $M_{j}$ adjacent miners and inserts the collected data into a contending block. Here, if the collected data is equal to or bigger than *N*th, the miner begins the block generation process. However, due to the processing time required for the local model learning of each edge node, fewer than *N*th nodes or miners can transmit the data. In this case, $m_{j}$ would not be able to collect *N*th or more data. In addition, queueing or transmission delays may cause $m_{j}$ to not collect enough data. Furthermore, $m_{j}$ may fail to receive signals due to the vulnerability of the wireless channel between $m_{j}$ and $n_{j,i}$ or $m_{j,k}$. In these cases, when $m_{j}$ fails to collect sufficient data, the miner is not involved in the block generation process; only the other miners participate in the process.

### 3.2. Jamming Attack by Malicious Miners

In this study, it was assumed that a malicious miner group participates in BDFL to make malicious blocks that are included in the mainstream while interfering with other normal nodes or miners using wireless jamming attacks. Notably, the objective of the malicious miner group is not to impact model aggregation by altering local model data. Instead, their aim is to affect the global model disseminated through the BDFL system by inserting malicious blocks into the blockchain system. We denoted a set of malicious miners, called attackers, as A where $A=\{a_{1},a_{2},\cdots ,a_{\left|A\right|}\}$. Attackers can propagate jamming signals; therefore, the outage probability of transmissions between normal nodes in N and M increases. Here, we assumed that the attackers perform reactive jamming, in which jamming signals are propagated when the transmitted signals are detected. Reactive jamming has a lower detection probability and higher power efficiency compared to continuous jamming.

Under jamming attacks by attackers, the received signal from $n_{j,i}$ at $m_{j}$ is represented as follows:

(1) $$y_{n_{j,i},m_{j}}=h_{n_{j,i},m_{j}}\sqrt{p_{n_{j,i}}}x_{n_{j,i}}+I\left(A_{n_{j,i}}\right)+z_{m_{j}},$$

where $h_{n_{j,i},m_{j}}$ is the channel gain between $n_{j,i}$ and $m_{j}$, $p_{n_{j,i}}$ is the transmission power of $n_{j,i}$, and $z_{m_{j}}$ is the white Gaussian noise with variance $\sigma^{2}$ at $m_{j}$. $I(A_{n_{j,i}})$ is the sum of strong interference from members of the attacker group located in the communication range of $n_{j,i}$ to detect the transmitted signal and reactively attack the corresponding signal. Here, $A_{n_{j,i}}$ is a set of attackers that cause $I(A_{n_{j,i}})$. When $m_{j,k}$ receives locally updated parameters from associated nodes, it broadcasts the received parameters to adjacent other miners, including $m_{j}$ for performing unbiased FL and accelerating the learning convergence. The received signal at $m_{j}$, which is transmitted from $m_{j,k}$, is represented as follows:

(2) $$y_{m_{j,k},m_{j}}=h_{m_{j,k},m_{j}}\sqrt{p_{m_{j,k}}}x_{m_{j,k}}+I\left(A_{m_{j,k}}\right)+z_{m_{j}},$$

where $h_{m_{j,k},m_{j}}$ is the channel gain between $m_{j,k}$ and $m_{j}$, $p_{m_{j,k}}$ is a transmission power of $m_{j,k}$, and $I(A_{m_{j,k}})$ is the sum of the strong interference from members of the attacker group located in the communication range of $m_{j,k}$ to detect the transmitted signals and reactively attack the corresponding signal. Here, $A_{m_{j,k}}$ is a set of attackers that cause $I(A_{m_{j,k}})$. In this study, it was assumed that signals transmitted from other nodes or miners do not interfere with the current signal due to the adopted transmission protocol for BDFL even if $m_{j}$ receives some interference signals that are negligibly small compared to strong jamming signals.

**Figure 2:**
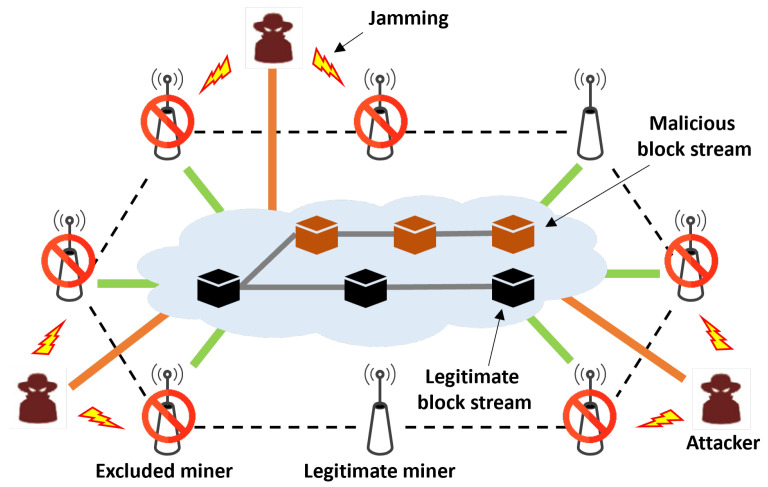
Blockchain-based decentralized federated learning with malicious miners in a wireless network.

Figure 2 shows the BDFL process in a wireless network comprising edge nodes, normal miners, and attackers. As shown in Figure 2, attackers launch a jamming attack on nearby miners, which disrupts their data receiving and prevents them from collecting enough data. These interrupted miners have difficulty collecting data, and some miners do not proceed with the block generation process. In this case, the number of attackers can be more significant than that of the normal miners, and attackers have a higher chance of successfully computing the hash value before the normal miners. Accordingly, the malicious block stream created by the attacker group can become mainstream. Section 4 presents a detailed explanation of malicious block insertion.

## 4. Jamming Attacks on Blockchain-Based Decentralized FL in Wireless Networks

In the BDFL system, blocks are verified by a proof-of-work, and one stream can be maintained through the consensus, such as the longest chain consensus adopted in the Bitcoin protocol. In the longest chain consensus, if two or more different streams are connected to one block for reasons such as network delay, and a chain fork occurs, only one stream is maintained with the longest chain as the mainstream. In this study, the longest chain consensus was adopted to maintain the mainstream.

Although the blockchain system is more reliable than the existing centralized system, there remains a risk of the mainstream being manipulated by attackers. The manipulated blocks are included in the mainstream when the following conditions are satisfied:

(i): The number of blocks generated by normal miners is greater than the number of transfer confirmations.
(ii): The number of blocks in the manipulated stream is greater than the number of blocks in the normal stream.

The normal transaction is completed when condition (i) is satisfied, whereas the manipulated stream becomes mainstream if condition (ii) is also satisfied by the longest chain consensus. Notably, the attack success probability depends not only on the computing power of normal and malicious miners but also on the stability of radio transmissions in a BDFL-based wireless network.

If normal miners have difficulty collecting data because of a jamming attack, there is a high probability that malicious miners can insert manipulated blocks into the mainstream. This is because the total computing power of the normal miners decreases, and as a result, a proportion of the normal miner group’s computing power decreases compared to the malicious miner group’s when miners who did not collect enough data due to reception failure drop out of the block mining process. In other words, the victim miner will not be able to collect enough data and will be left out of the mining process when wireless signals transmitted from edge nodes to the victim miner are detected and reactively interfered by attackers with jamming capability. Here, the attacker observes the target channel and immediately radiates a jamming signal when a target signal is detected for performing the reactive jamming attack. In this case, the number of blocks created by normal miner groups decreases, and it is possible for the attacker group to generate a longer block stream and turn this stream into the mainstream more easily when the jamming attack is successfully performed. In the following section, we analyze the attack success probability of malicious block insertion.

### 4.1. Miner Outage Probability

Under the assumption that the normal miners and edge nodes transmit data while avoiding signal collision, the SINR at miner $m_{j}$ from the associated node $n_{i}\in N_{j}$ is expressed as follows:

(3) $$\gamma_{n_{i},m_{j}}=\frac{p_{n_{i}}{\left|h_{n_{i},m_{j}}\right|}^{2}}{I\left(A_{n_{j,i}}\right)+\sigma^{2}}.$$

With simple data transmission in the Rayleigh fading channel, the outage probability at miner $m_{j}$ from node $n_{i}$ is represented as follows [32]:

(4) $$\begin{array}{cc} P^{out}\left(n_{i},m_{j}\right) & =P(\gamma_{n_{i},m_{j}}<\gamma_{th})\\ & =1-\exp\left(-\left(\frac{1}{{|h_{n_{i},m_{j}}|}^{2}}\right)\frac{\left(2^{2R_{t}}-1\right)}{\gamma_{n_{i},m_{j}}}\right), \end{array}$$

where γth is the SINR threshold and $R_{t}$ is the target probability. Similarly, the SINR and the corresponding outage probability from miner $m_{k}$ to $m_{j}$ are expressed as follows:

(5) $$\gamma_{m_{k},m_{j}}=\frac{p_{m_{k}}{\left|h_{m_{k},m_{j}}\right|}^{2}}{I\left(A_{m_{j,k}}\right)+\sigma^{2}},$$

(6) $$\begin{array}{cc} P^{out}\left(m_{k},m_{j}\right) & =P(\gamma_{m_{k},m_{j}}<\gamma_{th})\\ & =1-\exp\left(-\left(\frac{1}{{|h_{m_{k},m_{j}}|}^{2}}\right)\frac{\left(2^{2R_{t}}-1\right)}{\gamma_{m_{k},m_{j}}}\right). \end{array}$$

Here, the interference terms in Equations (3) and (5) toward miner $m_{j}$ are represented as follows:

(7) $$I\left(A_{n_{j,i}}\right)=\sum_{a_{i}\in A_{n_{j,i}}}p_{a_{i}}\;{|h_{a_{i},m_{j}}|}^{2},$$

(8) $$I\left(A_{m_{j,k}}\right)=\sum_{a_{k}\in A_{m_{j,k}}}p_{a_{k}}\;{|h_{a_{k},m_{j}}|}^{2}.$$

Note that $A_{n_{j,i}}$ and $A_{m_{j,k}}$ are the sets of attackers located in the communication range of $n_{i}$ and $m_{k}$ trying to transmit data to the miner $m_{j}$, as defined in Section 3.2.

Let $S_{m_{j}}$ be a set of normal miners/nodes that successfully transmit data to $m_{j}$. Then, $S_{m_{j}}$ can be represented as follows:

(9) $$\begin{array}{cc} S_{m_{j}}= & \{s_{j}|n_{i}\in N_{j}\mathrm{with}\;\mathrm{probability}\;\left(1-(4)\right),\\ \; & m_{j,k}\in \left(M\setminus m_{j}\right)\;\mathrm{with}\;\mathrm{probability}\;\left(1-(6)\right). \end{array}$$

Because miner $m_{j}$ does not perform the mining process when $|S_{m_{j}}|<N_{th}$, the set of miners participating in BDFL (i.e., $\hat{M}$) is represented as follows:

(10) $$\hat{M}=\{m_{j}|m_{j}\in M,\;|S_{m_{j}}|\geq N_{th}\}.$$

### 4.2. Attack Success Probability of Malicious Block Stream Generation

In this section, we describe the attack success probability when the set of miners participating in the blockchain process is denoted $\hat{M}$ instead of M. Here, the probability of (i) and (ii) are $P^{i}\left(\hat{M},A\right)$ and $P^{\mathrm{ii}}\left(\hat{M},A\right)$, respectively; therefore, the probabilities $P^{i}\left(\hat{M},A\right)$ and $P^{\mathrm{ii}}\left(\hat{M},A\right)$ can be calculated using the number of generated blocks. The number of blocks generated by normal miners in $\hat{M}$ and attackers in A is affected by the computing power of each group, and $B_{\hat{M}}$ and $B_{A}$ are the number of normal blocks and the number of malicious blocks originating from $\hat{M}$ and A, respectively. $f^{c}\left(\cdot \right)$ is the function of the computing power normalized by the total computing power sum of both normal miners and attackers in the networks; therefore, the probability $P^{i}\left(\hat{M},A\right)$ is calculated as follows [33]:

(11) Pi(M^,A)=BM^+BA−1BM^−1(fc(M^))BM^(fc(A))BA.

The probability $P^{\mathrm{ii}}\left(\hat{M},A\right)$ is calculated as follows [34]:

(12) $$\begin{array}{cc} \; & P^{\mathrm{ii}}\left(\hat{M},A\right)=\\ \; & \left\{\begin{array}{c} {\left(\frac{f^{c}\left(A\right)}{f^{c}\left(\hat{M}\right)}\right)}^{B_{\hat{M}}-B_{A}+1},\;f^{c}\left(\hat{M}\right)>f^{c}\left(A\right),\;B_{\hat{M}}\geq B_{A}\\ 1,\;\;\;\;\;\;\;\;\;\;\;\;\;\;\;\;\;\;\;\;\mathrm{otherwise} \end{array}\right\}. \end{array}$$

Using the probabilities calculated by Equations (11) and (12), the attack success probability $P^{att}\left(\hat{M},A\right)$ is calculated as follows:

(13) Patt(M^,A)=∑BA=0∞Pi(M^,A)Pii(M^,A)=1−∑BA=0BM^BM^+BA−1BM^−1(fc(M^))BM^(fc(A))BA×1−fc(A)fc(M^)BM^−BA+1,iffc(M^)>fc(A)1,iffc(M^)≤fc(A).

Equation (13) describes the probability that malicious blocks generated by attackers will be included in the mainstream. The malicious blocks included in the mainstream cause the malfunction or performance degradation of wireless nodes in the networks. Therefore, Equation (13) equals 1 when the computing power sum of the normal miners participating in BDFL is less than the computing power sum of the attackers.

In general, attackers generally increase high-cost computing power to increase the attack success probability. However, in a wireless BDFL system, attackers with jamming capability can perform jamming attacks instead of increasing computing power to insert malicious blocks into the blockchain system. As interference from attackers increases, normal miners find it difficult to participate in BDFL, as represented by Equations (9) and (10). Because $\hat{M}$ depends on the outage probabilities as calculated by Equations (4) and (6), the probability calculated by Equation (13) is eventually affected by the jamming success probability. Hence, without a significant increase in the computing power sum of attackers for block mining, that is $f^{c}\left(A\right)$, the probability calculated by Equation (13) could be increased by attackers with jamming capability.

It is worth nothing that the analysis of jamming attacks in the wireless BDFL system presented in this paper is not limited to FL applications and can be extended to the analysis of block insertion attempts by adversary groups with jamming capabilities in general wireless blockchain systems. Considering the ongoing researches in recent times concerning jamming attacks in wireless blockchain systems [35], this paper, which deals with attacks involving intentional selective jamming for block insertion rather than naive jamming, may have the potential to make a valuable contribution to such research. However, a detailed analysis of this matter falls outside the scope of this paper, so in this paper, we have limited our discussion to presenting its potential applicability.

### 4.3. Countermeasures

BDFL operating in a wireless network cannot be free from jamming attacks because of the inherent characteristics of the wireless channel. Naive countermeasures against jamming attacks include anti-jamming techniques like frequency hopping spread spectrum or direct sequence spread spectrum, which aim to evade channels where jamming signals are present or to prevent detection by jammers. However, these countermeasures may become ineffective if a miner with prior knowledge of the communication protocol participates in the attack. Security rules can be used to prevent the participation of malicious miners; however, because the participating miners may have different purposes after approval, countermeasures are required to respond to such threats. In other words, it is necessary for a blockchain system to detect and hinder block insertion attacks by malicious miner groups with jamming capability. One possible approach is to detect abnormalities by increasing the likelihood of an attack’s existence through a dedicated security channel, especially when an edge node identifies persistent transmission failures to the miner in the corresponding channel. Then, miners who have reported this can check the communication or channel status with other nodes and determine whether there is an attack. For example, under this process, if there is a notable discrepancy in channel conditions among neighboring miners, those with unusually better channel conditions may be subject to suspicion. Then, measures such as imposing restrictions on the block insertion activities of these miners can be considered. On the other hand, by pre-assessing the computing power of miners, the blockchain system can identify anomalies by comparing the block generation rate of specific miners against their computing capabilities, flagging rates that are disproportionately high or low. As a countermeasure that can reduce the effectiveness of the attack, if the number of successful hash calculations for each miner or a randomly selected group of miners is higher or lower than a certain threshold within a certain time window, the block transmitted at this time is ignored and the next miner’s block selected. In this case, since miner groups are randomly selected and partitioned, it becomes easier to detect the presence of an attack if a malicious miner group attempts to disrupt the mining participation of nearby miners. Additionally, periodically updating the grouping and refraining from disclosing group designation information to miners can also prevent targeted attacks.

## 5. Performance Evaluation

In this section, we present the results of MATLAB-based simulations designed to evaluate the impact of jamming on transmission outage and the success probability of a block insertion attack in a wireless BDFL system. In the network, the normal miners are evenly distributed across the area in a grid pattern. Normal nodes are uniformly distributed in the network, and each node is associated with the closest normal miner to achieve high received signal strength. Attackers are randomly distributed in the network, and they reactively propagate jamming signals when the data signals of local model parameters from miners or edge nodes participating in BDFL are detected. For the simulation, we assumed that the computing powers of miners and attackers were the same.

The simulation was implemented in MATLAB, and the simulation parameters are listed in Table 1. We assumed a network space of 100 × 100 $m^{2}$ for the simulations. Transmissions were performed at a carrier frequency of 2.4 GHz and attenuated with a pathloss exponent of 3 under the Rayleigh fading channel. The transmission power of normal nodes, normal miners, and attackers was equally 10 dBm for each. The target probability $R_{t}$ calculated by Equations (4) and (6) was set at $R_{t}\in \left\{0.5,1.5\right\}$ bps/Hz. As the target rate increases, the required SINR for successful transmission increases, and eventually, the outage probability increases, as calculated by Equations (4) and (6). Attackers with jamming capability can employ either omnidirectional or beamforming jamming. Depending on the jamming strategy employed by attackers, the amount of mean interference toward normal nodes or miners calculated by Equations (7) and (8) is then determined. For beamforming jamming, the beamforming gain is added toward the channel gain toward the target. In the simulation, we assumed 10 dBi beamforming gain for jamming signals toward the attack target, as followed by [28]. Note that the beamforming gain of the jamming signals can be increased by narrowing the beam width. However, as the beam width narrows, the number of victim miners or normal nodes decreases depending on the network topology. In the simulation, we assumed a grid and uniform distribution of miners and normal nodes, respectively, to consider common network scenarios. Thus, we set the attackers to perform beamforming jamming on the nearest miner; otherwise, we performed omnidirectional jamming attacks.

**Figure 3:**
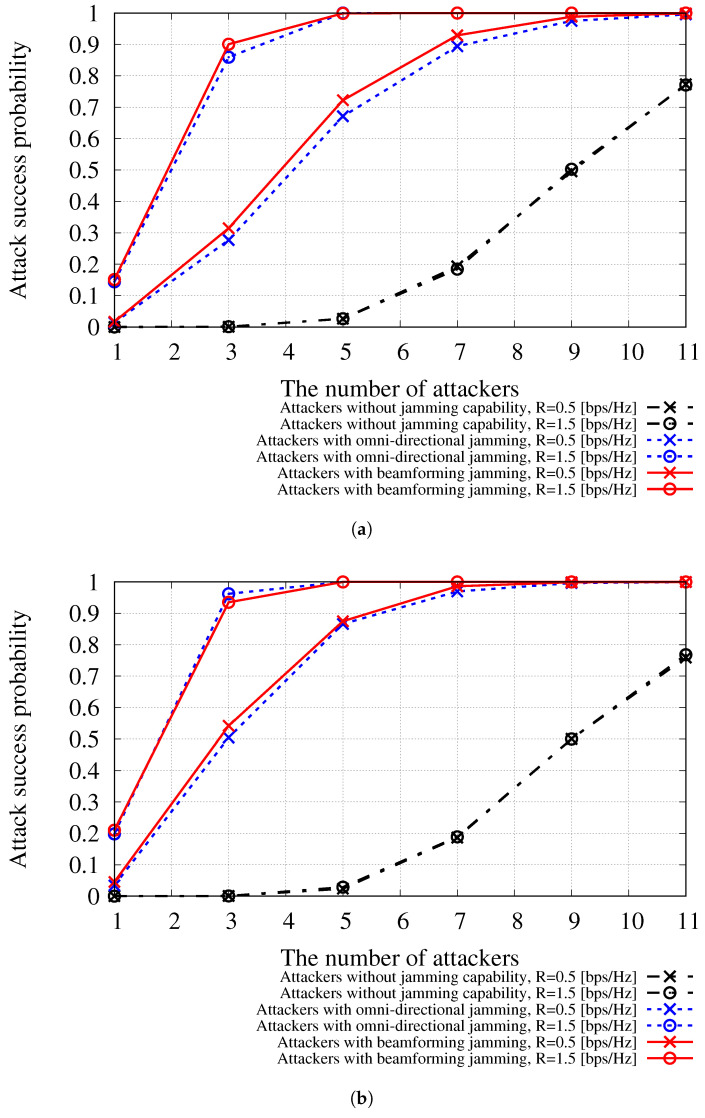
Attack success probability when |M|=9 and |N|=45 with regard to the number of attackers. (**a**) Attack success probability, *N*th = 3; (**b**) attack success probability, *N*th = 7.

Figure 3 shows the attack success probability with respect to the number of attackers when |M|=9 and |N|=45. As shown in Figure 3a, when attackers without jamming capability are deployed in the network, the attack success probability depends strictly on the number of attackers. Note that we assumed that the computing power of miners and attackers is the same for block generation; thus, the average attack success probability is 0.5 when there are nine attackers in the network. When attackers with jamming capability are deployed, they can achieve a higher attack success probability even when the number of attackers is less than the number of miners. As shown in Figure 3, beamforming jamming achieved slightly higher attack success probabilities than omnidirectional jamming. The simulation results imply that there could be significant performance degradation in BDFL-based wireless services when attackers have jamming capability. The simulation results also show that an increase in target probability may lead to a higher attack success probability because the outage probabilities for transmissions between non-attacker nodes such as normal miners or normal nodes increase. Figure 3b shows the results when *N*th is increased to 7 compared to the results where *N*th = 3, as shown in Figure 3a. In this case, because the miners need to gather more data to participate in BDFL, as calculated by Equations (9) and (10), higher outage probabilities are introduced compared to the results shown in Figure 3a.

**Figure 4:**
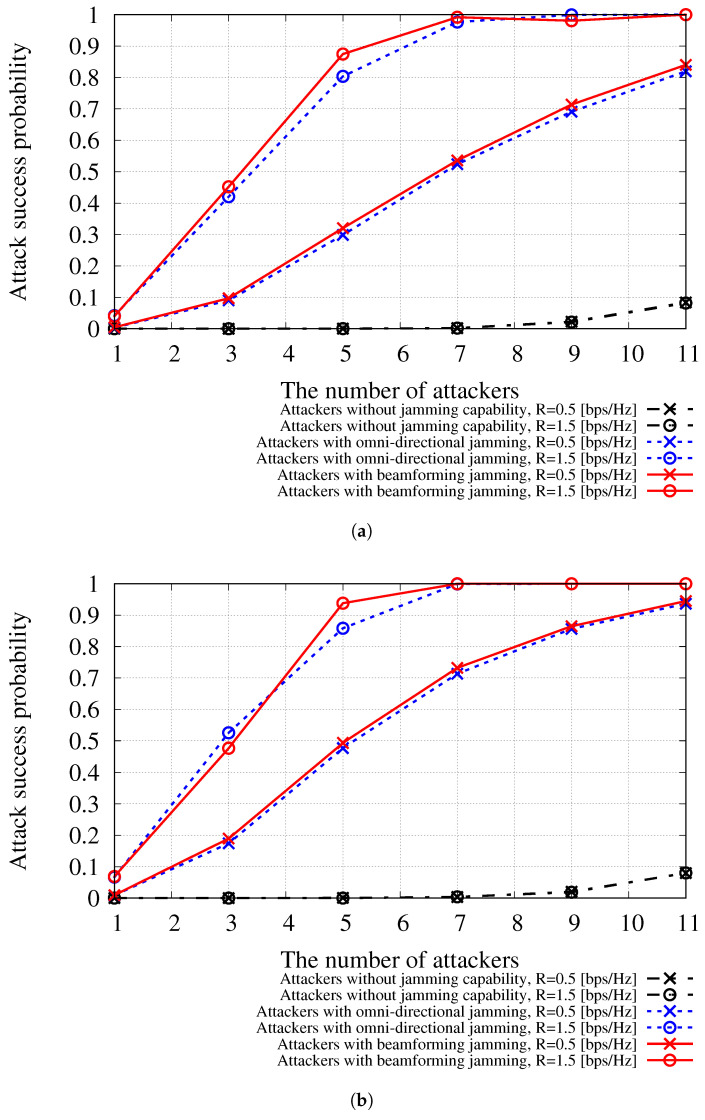
Attack success probability when |M|=16 and |N|=80 with regard to the number of attackers. (**a**) Attack success probability, *N*th = 3; (**b**) attack success probability, *N*th = 7.

Figure 4 shows the attack success probability when |M|=16 and |N|=80. The results show that the attack success probabilities of attackers without jamming capability significantly decrease compared to the results shown in Figure 3. This is because the number of normal miners increases and the attackers need higher computing power for block generation to make malicious blocks become mainstream. However, if the attackers can effectively interfere with other nodes by jamming signals, they will achieve a higher attack success probability.

**Figure 5:**
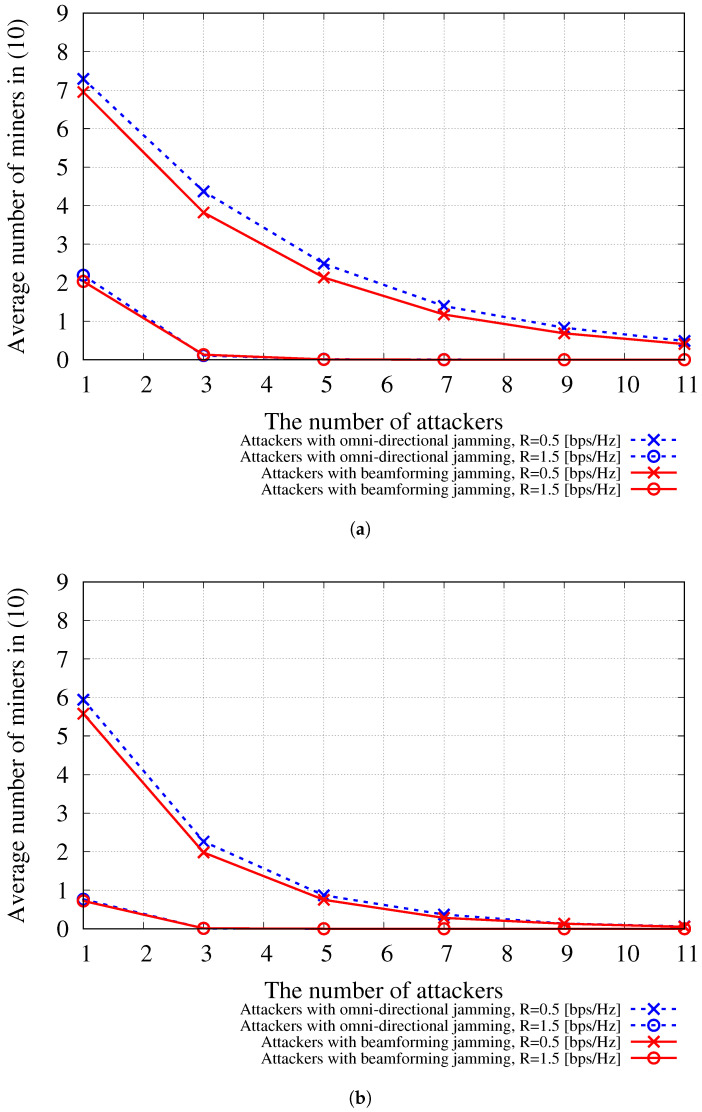
Average number of normal miners actually participating in BDFL when |M|=9 and |N|=45 with regard to the number of attackers. (**a**) Average normal miners ($|\hat{M}|$), *N*th = 3; (**b**) Average normal miners ($|\hat{M}|$), *N*th = 7.

Figure 5 shows the average number of miners participating in the BDFL calculated by Equation (10) with respect to the number of attackers with jamming capability when |M|=9 and |N|=45. As shown in the results, in some wireless network scenarios, attackers with jamming capability can significantly impair the BDFL service by disrupting miners’ participation.

Using the simulation results, we verified the impact of jamming attacks on the BDFL system when an attacker group attempts to insert malicious blocks into the mainstream. Because the number of participating miners is reduced when miners fail to collect enough data because of a jamming attack, the attack success probability increases as the number of attackers with jamming capability increases or the number of required *N*th increases. Therefore, it is necessary to consider the capability of attackers when designing a BDFL system in a wireless network and setting the parameters. To respond to the above attack, each miner needs to be able to adjust the *N*th value adaptively by considering the number of connected edge nodes and the channel environment. In addition, the blockchain mechanism should be able to identify abnormalities and devise countermeasures to deal with them.

## 6. Conclusions

In this study, we investigated the impact of jamming attacks on BDFL in wireless networks. In general, to increase the attack success probability of malicious block insertion on blockchain systems, attackers must increase the mining computational power to generate more blocks than normal miners. However, when the attack is performed in a wireless network where the transmission channel is vulnerable to interference signals, attackers can increase the success probability with less computing power by failing to receive signals from normal miners during a jamming attack. Therefore, service providers should pay more attention to wireless channels when employing blockchain systems for FL mechanism. In this study, we studied the outage and attack success probabilities when attackers with jamming capability perform an attack to insert a malicious block into a BDFL system. Through various simulations, we evaluated the performance of the success probability of malicious block insertion and the participation rate of normal miners in the wireless BDFL system. Here, we also verified that a malicious miner group can increase the attack success probability by performing a jamming attack instead of significantly increasing high-cost computational power.

In future work, we intend to develop specific countermeasures against jamming threats that may arise in wireless systems adopting BDFL. We aim to develop an algorithm that can detect the existence of malicious miners and prevent block stream generation from being dominated by specific miner groups with jamming capability. In addition, our next study will address developing algorithms that consider potential vulnerabilities in the model aggregation process, as well as the integrity and confidentiality of aggregated model updates. Moreover, we plan to build and evaluate the performance of a system targeting specific protocols of blockchain and FL. Additionally, we aim to observe the performance changes of the system under various jamming techniques and assess the robustness of the system with the developed countermeasures.

## Figures and Tables

**Table 1:** Wireless channel-related parameters.

Parameter	Value
Carrier frequency	2.4 [GHz]
Pathloss exponent	3
Small-scale fading	Rayleigh fading
Transmission power	10 [dBm]
Beamforming jamming	10 [dBi]
Target probability	{0.5, 1.5} [bps/Hz]
Network area	100 × 100 [$m^{2}$]

## Data Availability

Data are contained within the article.
